# miR‐21‐5p/SMAD7 axis promotes the progress of lung cancer

**DOI:** 10.1111/1759-7714.14060

**Published:** 2021-07-12

**Authors:** Jinming Tang, Xu Li, Tianli Cheng, Jie Wu

**Affiliations:** ^1^ Department II of Thoracic Surgery, Hunan Cancer Hospital, The Affiliated Cancer Hospital of Xiangya School of Medical Central South University Changsha China; ^2^ Department I of Thoracic Medicine, Hunan Cancer Hospital, The Affiliated Cancer Hospital of Xiangya School of Medical Central South University Changsha China

**Keywords:** lung cancer, microRNA, miR‐21‐5p, SMAD7

## Abstract

**Background:**

Lung cancer is one of the most common malignant tumors threatening human health. The aim of this study was to investigate the function of miR‐21‐5p in lung cancer progression.

**Methods:**

We analyzed the expression levels of miR‐21‐5p in lung cancer tissues and cell lines. The qRT‐PCR and MTT assays were performed after transfection with miR‐21‐5p mimic, inhibitor and negative control into lung cancer cells.

**Results:**

Luciferase reporter assays showed miR‐21‐5p directly target SMAD7. The miR‐21‐5p inhibitor significantly suppressed lung cancer cell proliferation, invasion and migration. We found that SMAD7 was upregulated in lung cancer tissue. In addition, we found that SMAD7 inhibited lung cancer cell proliferation and miR‐21‐5p mimic damaged the inhibitory effect of SMAD7.

**Conclusions:**

miRNA‐21‐5p may promote cell proliferation, migration and invasion by spoiling SMAD7 expression in lung cancer cells.

## Graphical abstract

miR‐21‐5p directly targets SMAD7. miR‐21‐5p/SMAD7 signal controls lung cancer progression.

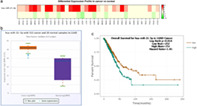

## INTRODUCTION

Lung cancer is one of the most common malignant tumors threatening human health.^1^ Although the treatment of lung cancer has made great progress, the mortality rate is increasing year by year.^2^ So far, little is known about the mechanism of lung cancer occurrence and development. The occurrence of lung cancer is a multifactor, multistage, multigene synergistic process which is related to the dysregulation expression of oncogenes and tumor suppressor genes.^3^, ^4^

Recent studies have revealed that some microRNAs (miRNAs) are involved in tumor formation. MicroRNAs are a class of non‐coding single‐stranded small molecule RNA with a length of 19 to 22 nucleotides that have been discovered in recent years.^5^ Mature miRNAs are retained in a functional complex and regulate gene expression by binding to the 3′‐untranslated region of target mRNA.^6^ MicroRNAs play a crucial role in many biological processes from embryonic development, differentiation, immune regulation, disease cause (including oncogenes activation and tumor suppressor genes inactivation) and senescence.^2^, ^7^ In recent years, more and more studies have shown that many miRNAs can be used as proto‐oncogenes or tumor suppressor genes, and play an important role in the occurrence and development of cancer. Among them, miRNA‐21 is the most interesting.

The coding gene of miRNA‐21 is located at 17q23.2, vacuolemembraneprotein‐1 (VMP1) coding region, the tenth intron of VMP1 gene. Studies have shown that some tumors have genomic 17q23.2 amplification mutations, such as breast and prostate cancer, and Hodgkin's lymphoma.^8^ Overexpression of miRNA‐21‐5p has been confirmed in various cancers, such as breast, prostate, and gastric cancer.^9^, ^10^, ^11^ The clinical significance of miRNA‐21‐5p expression in human cancer has been previously reported.^12^ miRNA‐21‐5p is involved in cell proliferation, differentiation, and apoptosis, and is therefore closely related to tumor growth, invasion, and metastasis. Yan et al. reported that miRNA‐21‐5p overexpression is associated with specific clinical pathological characteristics of breast cancer, advanced tumor stage, lymph node metastasis, and patient survival.^13^ Although many studies have reported that the expression level of miRNA‐21‐5p is significantly increased in tumor cells and has oncogenic activity, because microRNA has several or even dozens of target genes, the mechanism of cell biological behavior changes caused by miRNA‐21 has not yet been fully elucidated. The relationship between miRNA‐21‐5p expression and lung cancer has not been fully elucidated, and more detailed mechanism studies are needed to elucidate its complex biological role.

Studies have shown abnormal expression of SMAD7 in various types of tumors such as breast, gastric, pancreatic and prostate cancers. In colorectal cancer, SMAD7 protein expression is increased and its overexpression is negatively correlated with the survival rate of colorectal cancer patients.^14^ Kleeff et al. report that the SMAD7 expression level in pancreatic cancer is higher by comparison with that of the normal pancreas.^15^ The pancreatic cancer cells transfected with SMAD7 have been found to have increased malignancy and enhanced tumorigenic ability in nude mice, suggesting that abnormal expression of SMAD7 will contribute to tumor development.^16^ However, some studies have shown different results. It has been reported that transfection of SMAD7 antisense gene could block the growth promotion of TGFbeta.^17^ At present, there are few studies on the role of SMAD7 in the occurrence, development, invasion and metastasis of lung cancer.

## METHODS

### Cells and reagents

The human lung cancer cell lines A549, H460, H1299 and normal human bronchial epithelial cell line (HBE) were purchased from the Shanghai Cell Bank of Chinese Academy of Sciences. All cells were cultured in RPMI1640 cell culture medium containing 100 ml/L fetal bovine serum, 100 U/ml penicillin, 100 g/ml streptomycin and 2 mmol/L glutamine at 37°C with 5% CO_2_ in full humidity.

### Transient transfection

Cells were counted and seeded on a 24‐well plate one day before transfection. RPMI1640 culture solution with no serum or antibiotics was diluted for transfection. miRNA‐21‐5p inhibitor, mimic and negative control were designed and synthesized by Biomic Biotechnology (USA). The sequence of primers are as follows: miRNA‐21‐5p sense chain of mimic: 5′‐UAGCUUAUCAGACUGAUGUUGA‐3′, antisense chain: 5′‐AACAUCAGUCAUACUAUU‐3′; miRNA‐21‐5p sense chain of inhibitor: 5′‐UCAACAUCAGUCUGAUACUA‐3′. The miRNA‐21‐5p inhibitor, the mimic and the negative control were mixed with the LipofectamineTM 2000 reagent, left at room temperature for 20 min, then added to the corresponding well cells, and incubated at 37°C in a 5% CO_2_ incubator for 4–6 h.

### Quantitative real‐time (qRT‐PCR)

Total RNA was extracted from lung cancer cells using TRIzol reagent (Life Technologies). The cell growth medium was removed and 1 ml of TRIzol reagent was added to each 10^5^ cells to lyse the cells. Then, 200 μl of chloroform was added for every 1 ml of TRIzol and incubated for 2 min. The sample was centrifuged at 12 000 × *g* at 4°C for 15 min. The aqueous phase containing RNA was collected, and the RNA was precipitated, washed, and dissolved. The miScript reverse transcription kit (Qiagen) was used for reverse transcription (RT) of microRNA, and the TaqMan universal PCR master mix was used to detect the expression of mature miRNA‐21‐5p.

### MTT

Cells were seeded in 96‐well plates with a density of 2000 cells/well in 100 μl medium. The plate was incubated for the appropriate period and then 10 μl of MTT (5 g/L) solution was added to each well of the plate. Incubation continued for 4 h, and the culture medium was carefully aspirated from the well. Then, 150 μl DMSO was added to each well and and the plate was shaken for 10 min to fully dissolve the crystals. The optical density value (OD) at 450 nm was detected using a microplate spectrophotometer.

### Transwell assay

The cell culture inserts were placed into the wells of the culture plate. The polycarbonate filter was either precoated with 50 μl of Matrigel for the invasion assay or uncoated for the migration assay. The cells were seeded into the upper chamber with a medium without FBS. The lower chambers were filled with culture medium with 10% FBS. After incubation for 48 h, the cells (noninvasive cells) on the upper surface of the insert were removed with a cotton swab. The invaded cells were stained with 0.1% crystal violet. The number of cells passing through the membrane in five different fields of view under a 100x light microscope were selected, and the average value calculated.

### Luciferase reporter assay

The wild‐type (wt) or mutated (mut) mir‐21‐5p binding sequences from SMAD7 3′UTR were cloned into the pGL3 basic vector. Cells seeded at a density of 50% were placed into a 12‐well culture plate. After 16 h of continuous culture, the luciferase reporter gene plasmid and pRL‐TK plasmid were transfected when the cell density was about 70%. According to the manufacturer's protocol, Lipofectamine 2000 was used to cotransfect the cells with mir‐21‐5p mimics, SMAD7 (wt) and SMAD7 (mut). The renilla luciferase was used as a loading control. The cells were harvested. Luciferase activity was analyzed using a dual luciferase reporting system (Promega). In brief, after cotransfection of the plasmid for 48 h, the medium was discarded and the cells were washed with PBS. The 12‐well plate was tilted and the remaining PBS aspirated. Then, 50 μl PLB was added to each well and shaken for 30 min on a shaker to ensure complete cell lysis. In a white opaque 96‐well plate, 10 μl supernatant and 100 μl luciferase assay reagent II was added to each well, and the luciferase intensity measured. After the test had been completed, we added 100 μl Stop&Glo reagent to each well, and measured the renilla luciferase activity.

### Statistical analysis

All experiments were performed independently at least three times. Statistical analysis was performed using a Student's *t*‐test or a one way ANOVA. All data is shown as mean+/− SEM.

## RESULTS

### miR‐21‐5p is upregulated in lung cancer and correlated with prognosis

**FIGURE 1:**
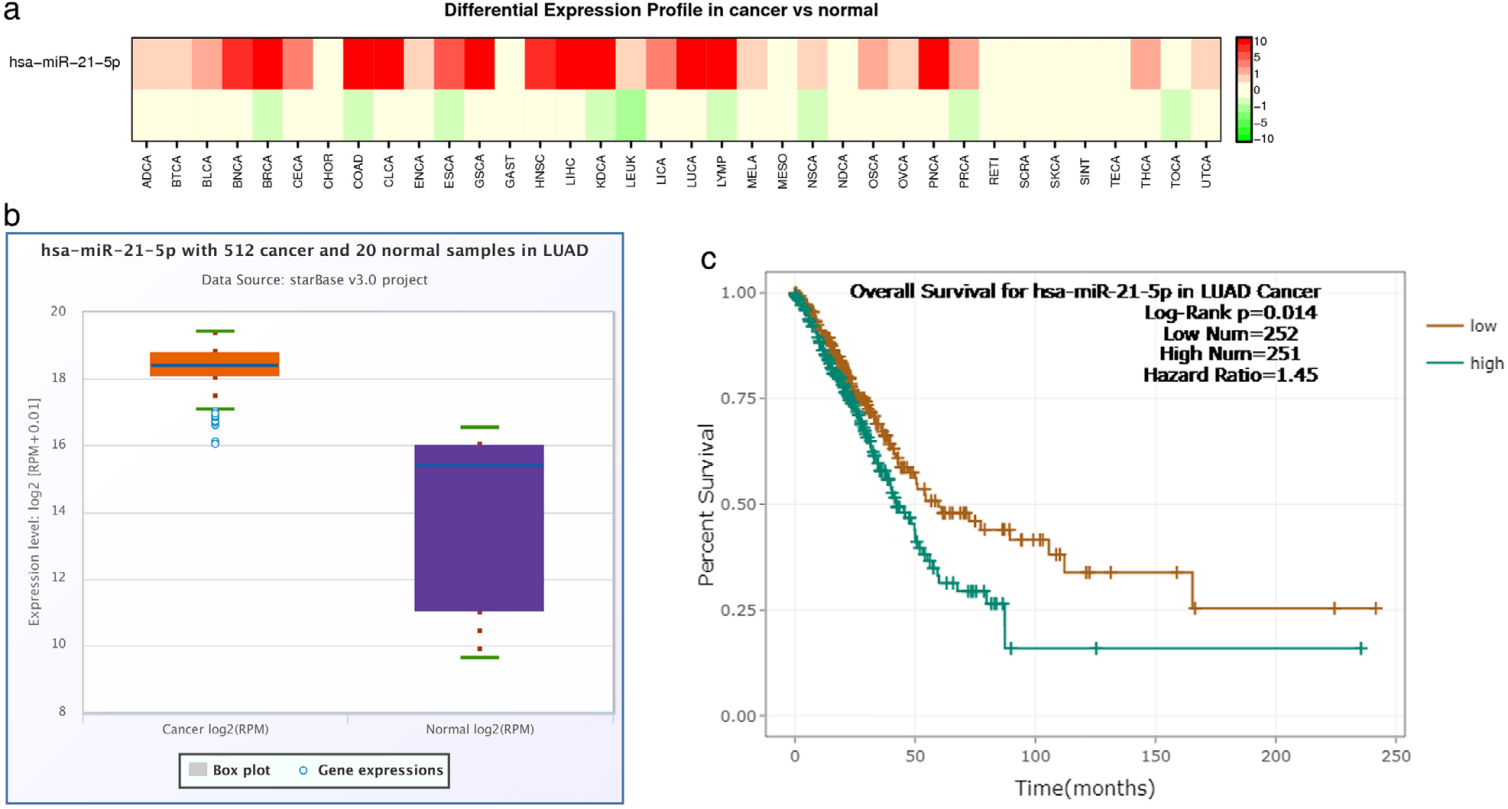
miR‐21‐5p is upregulated in lung cancer and is correlated with prognosis. (a) The pan‐cancer analysis of miR‐21‐5p differential expression. (b) High miR‐21‐5p expression in lung cancer compared with normal tissue. (c) Relationship between miR‐21‐5p expression level and overall survival of patients with lung cancer

We performed a pan‐cancer analysis of miR‐21‐5p expression using dbDEMC 2.0 database (https://www.biosino.org/dbDEMC/index). A heat map showed that the expression level of miR‐21‐5p was significantly upregulated in lung cancer tissues (LUCA) (Figure 1(a)). In addition, the expression of miR‐21‐5p between lung cancer and normal lung tissue was initially analyzed using the starBase v2.0 database (http://starbase.sysu.edu.cn/) (Figure 1(b)). In addition, the relationship between miR‐21‐5p expression and the prognosis of lung cancer patients was analyzed. The starBase database analysis results showed the survival rate of lung cancer patients with low expression of miR‐21‐5p was better than that of the patients with high expression of miR‐21‐5p (Figure 1(c)).

### Function of miR‐21‐5p in lung cancer cells

**FIGURE 2:**
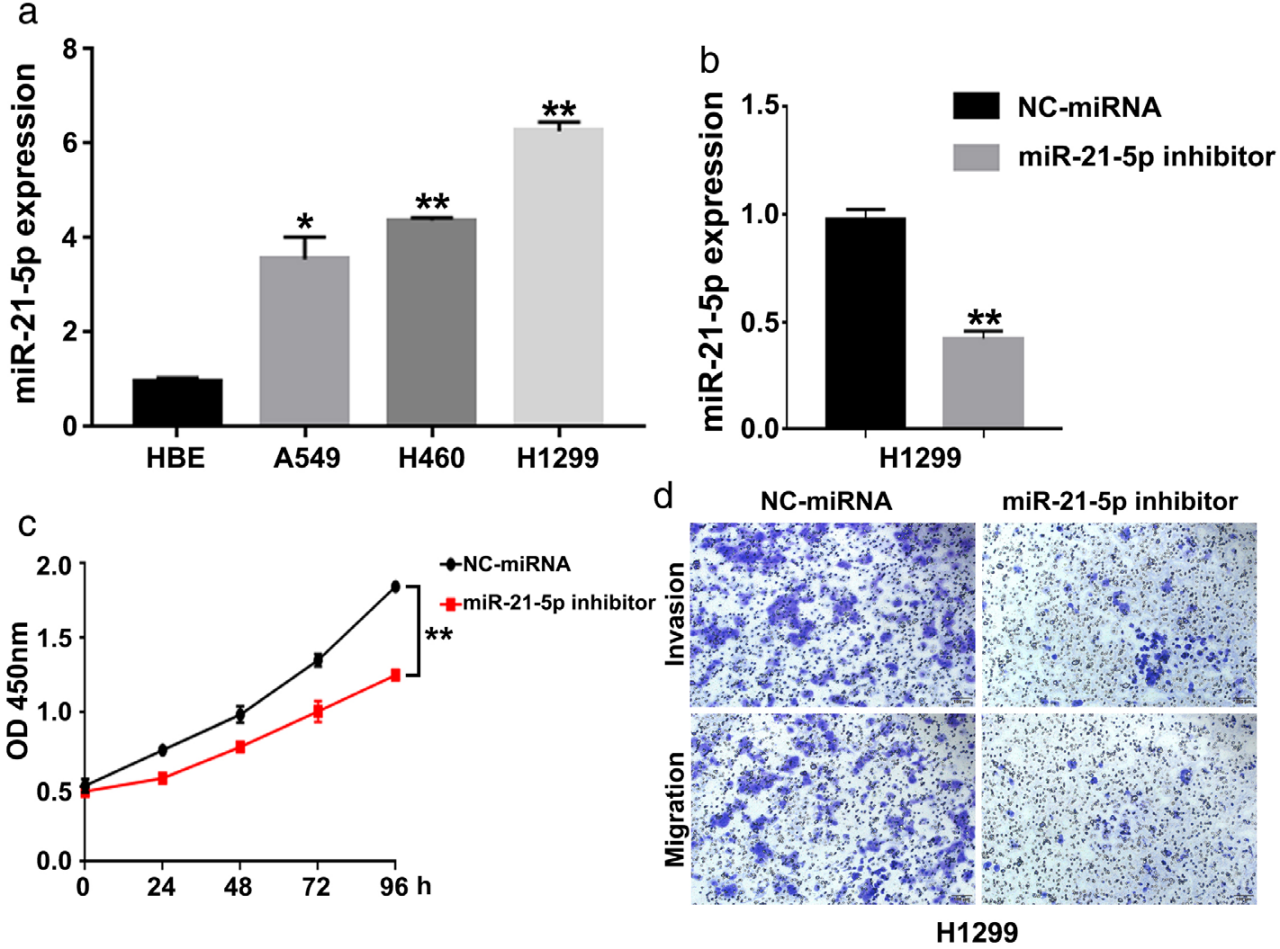
The function of miR‐21‐5p in lung cancer cells. (a) The expression level of miR‐21‐5p in lung cancer cell lines. (b) miR‐21‐5p expression in H1299 cells was measured after transfection with miR‐21‐5p inhibitor. (c) Cell proliferation was detected in H1299 cells after transfection with miR‐21‐5p inhibitor. (d) Transwell assay was performed in H1299 cells after transfection with miR‐21‐5p inhibitor. **p < 0.01, *p < 0.05, one‐way ANOVA for multiple comparisons in a Student's *t*‐test was used in (b) and (c)

RT‐qPCR results showed that miR‐21‐5p expression in lung cancer cell lines (A549, H460, H1299) was significantly higher than that in normal bronchial epithelial cells (HBE) (*p* < 0.05). As shown in Figure 2(a), the expression level of miR‐21‐5p was the highest in H1299 cells. The miR‐21‐5p inhibitor was transfected into H1299 cells. The results of RT‐qPCR showed that miR‐21‐5p inhibitor significantly reduced the expression level of miR‐21‐5p (Figure 2(b)). The MTT assay showed that miR‐21‐5p inhibitor inhibited the proliferation of lung cancer cells (Figure 2(c)). In addition, a transwell assay was used to compare the effect of miR‐21‐5p expression on the migration and invasion ability of lung cancer cells. The data showed that miR‐21‐5p inhibitor inhibited the invasion and migration of lung cancer cells (Figure 2(d)).

### miR‐21‐5p targets SMAD7 in lung cancer cells

**FIGURE 3:**
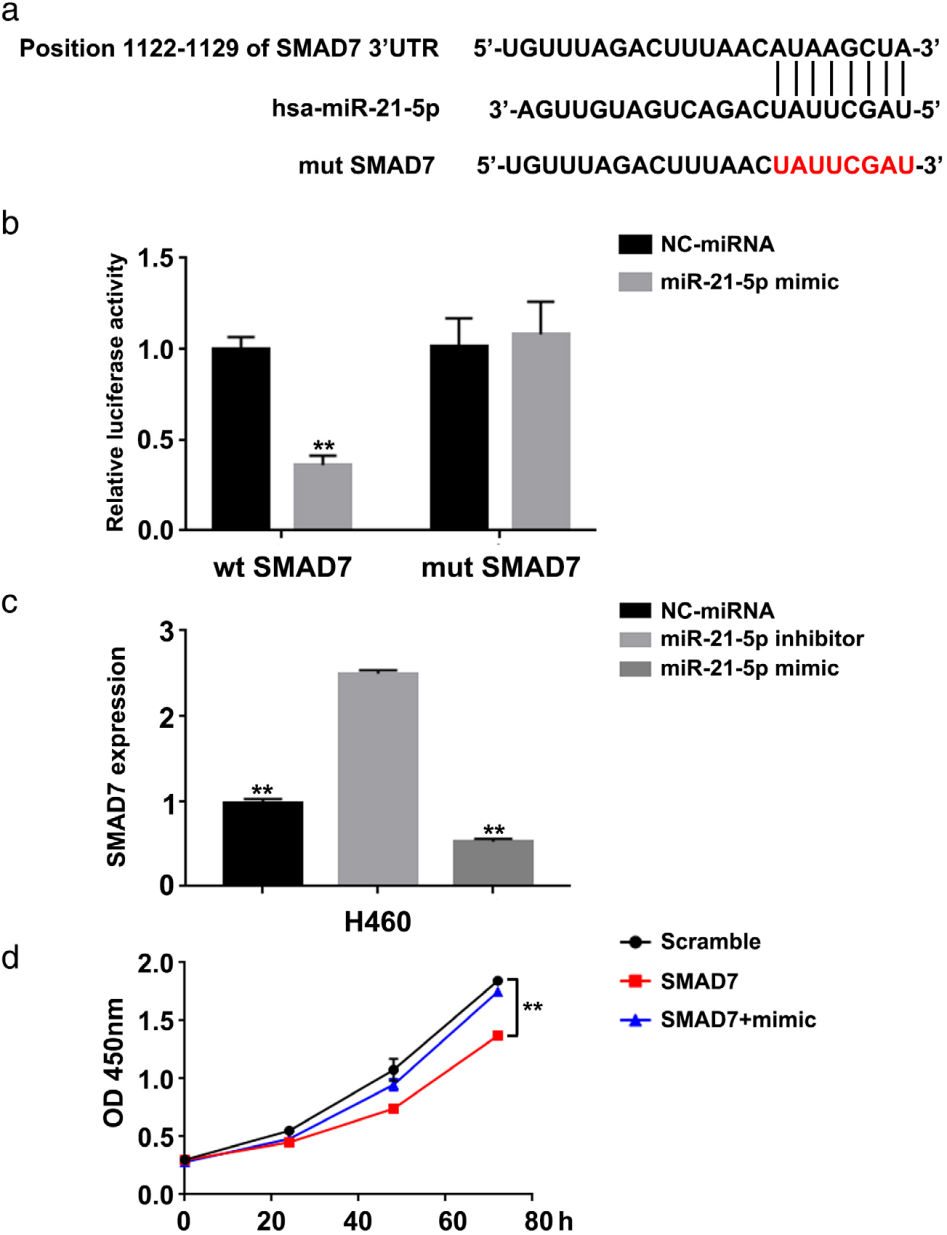
The miR‐21‐5p targets SMAD7 in lung cancer cells. (a) Predicted binding site between miR‐21‐5p and 3′‐UTR of SMAD7. (b) Luciferase activity in H460 cells transfected with wild‐type or mutation SMAD7 and miR‐21‐5p mimic or NC‐miRNA. (c) SMAD7 mRNA expression was measured in H460 cells. (d) Cell proliferation was detected in H460. **p < 0.01, one‐way ANOVA for multiple comparisons in (b), (c) and (d)

To identify miR‐21‐5p target genes involved in lung cancer cell behavior, we searched the Targetscan database. The results showed that there was a complementary sequence with miR‐21‐5p on the 3′UTR end of SMAD7 mRNA (Figure 3(a)). Renilla‐luciferase reporter assay was performed to determine whether SMAD7 was a direct target of miR‐21‐5p. We cloned the full length 3′UTR sequence of SMAD7 mRNA containing wild‐type or mutant miR‐21‐5p binding sequence in the dual luciferase vector. Dual‐luciferase reporter assay showed that miR‐21‐5p mimic inhibited luciferase activity of the SMAD7 reporter gene (Figure 3(b)). In addition, we found that miR‐21‐5p mimic could reduce the expression level of SMAD7 mRNA, while miR‐21‐5p inhibitor could increase the expression level of SMAD7 mRNA (Figure 3(c)). These results suggest that SMAD7 is a direct target of miR‐21‐5p in lung cancer cells. The miR‐21‐5p mimic could abrogate the inhibition of proliferation by ectopically expressing SMAD7 in SMAD7‐overexpressed CRC cells (Figure 3(d)).

### Expression level of SMAD7 in lung cancer tissues

**FIGURE 4:**
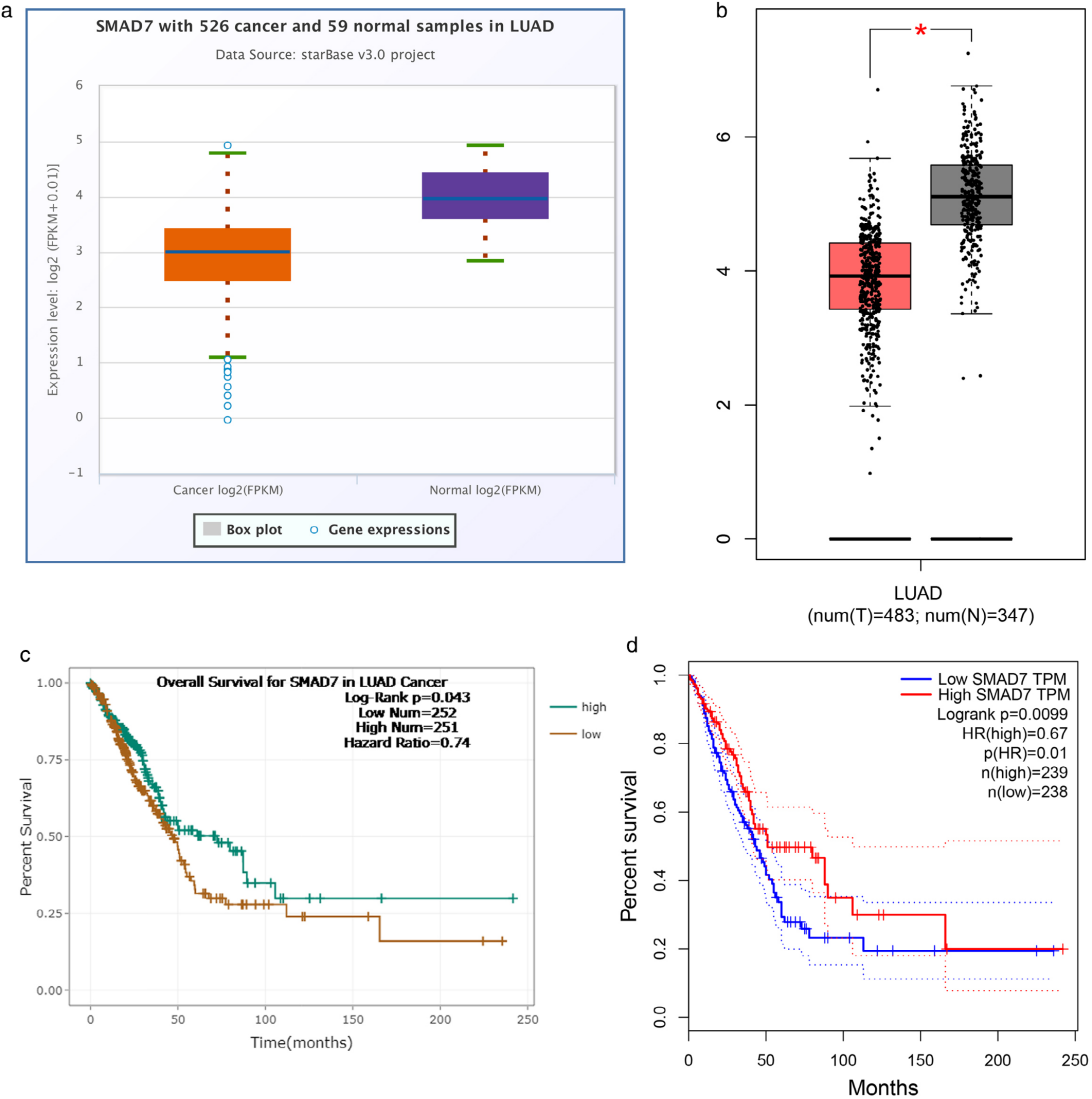
The expression level of SMAD7 in lung cancer tissues. (a) The expression level of SMAD7 in lung cancer and normal lung tissues using the starBase database. (b) The expression level of SMAD7 in lung cancer and normal lung tissues using the GEPIA database. (c) The StarBase database showed a relationship between SMAD7 expression level and prognosis of patients with lung cancer. (d) The GEPIA database showed a relationship between SMAD7 expression level and prognosis of patients with lung cancer

We analyzed SMAD7 expression in lung cancer tissues using the starBase and GEPIA databases. Statistical analysis of SMAD7 expression showed a reduction in lung cancer tissues compared to normal lung tissues (Figure 4(a) and (b)). In addition, according to the survival curves defined by high and low expression of SMAD7 in lung cancer patients, it was found that a high expression level of SMAD7 had an important effect on the overall survival of patients. The starBase and GEPIA databases showed that high levels of SMAD7 expression had a good effect on overall survival of patients (Figures 4(c) and (d)).

## DISCUSSION

Abnormal cell proliferation is an important feature of tumor biology, and miRNAs play an important role in regulating cell proliferation, differentiation, and apoptosis. Many studies have reported that miRNAs play a role of carcinogenic or tumor suppressor genes. Some miRNAs with carcinogenic effects can promote the occurrence of tumors by inhibiting the expression of tumor suppressor genes. Compared with normal tissues, the expression level of miRNAs in tumor tissues is significantly different from normal tissues, so these abnormally expressed miRNAs are expected to be important markers for tumor diagnosis, prognosis and treatment. Studies have shown that miRNA‐21‐5p expression in various tumor tissues is increased, such as in gastric, prostate, breast, pancreatic, cervical, and colon cancers.^18^, ^19^ Inhibiting the expression of miRNA‐21‐5p can reduce the proliferation of breast cancer cell lines and promote apoptosis of breast cancer cells by indirectly downregulating the antiapoptotic factor Bcl‐2. After inhibiting miRNA‐21‐5p expression with antisense oligonucleotides, activity of Caspase‐3 and Caspase‐7 increased, and apoptosis of glioblastoma cells increased significantly, which shows that miRNA‐21‐5p has an antiapoptotic effect and its abnormal expression inhibits expression of death‐related genes causing tumorigenesis. Seike et al. treated H3255 cell lines with *EGFR* mutation and H441 cell lines with wild‐type *EGFR* mutation with EGFR‐TKIs, and the miRNA‐21‐5p expression in these two cell lines was downregulated, indicating that the activated EGFR signaling pathway has a positive regulatory effect on miRNA‐21‐5p.^20^ In addition, several studies have confirmed that miRNA‐21‐5p can regulate tumor cell proliferation, migration and sensitivity to chemotherapy by acting on the tumor suppressor genes PTEN, MARCKS, PDCD4, and Cdc25A.^21^, ^22^, ^23^, ^24^ In summary, miRNA‐21‐5p is closely related to the occurrence and development of various tumors, and can be used for tumor diagnosis, treatment and prognosis, and has important clinical significance. However, due to the lack of in‐depth understanding of the transcriptional regulation mechanism of miRNA‐21‐5p target genes, the clinical application of miRNA‐21‐5p is limited. It can be expected that with further research, miR_21 will have broad application prospects in the diagnosis and treatment of tumors.

It has been previously reported that SMAD7 expression is reduced in cisplatin‐resistant lung cancer A549 cell lines.^25^ In this study, miRNA‐21‐5p mimics and inhibitors were transfected into H460 cells. The results of the qRT‐PCR assay showed that SMAD7 expression increased significantly in cells after transfecting miRNA‐21‐5p inhibitor; the expression of SMAD7 in cells was significantly reduced after transfection with miRNA‐21‐5p mimic.

In the MTT experiment, the cells transfected with SMAD7 had a significantly decreased OD 450 nm value compared to the scramble transfected cells. At the same time, there was no difference in the cells cotransfected with miRNA‐21‐5p mimic and SMAD7 with scramble. The above results suggest that miRNA‐21‐5p mimic may promote the proliferation of H460 cells by inhibiting SMAD7 expression.

In summary, miRNA‐21‐5p affects the migration and proliferation of lung cancer H1299 cells through targeted regulation of SMAD7 expression in lung cancer cells, suggesting that miRNA‐21‐5p is a potential target, which can be used for the intervention and prevention of the lung cancer process.

## CONFLICT OF INTEREST

No conflict of interest is reported.
